# Oral delivery of siRNA lipid nanoparticles: Fate in the GI tract

**DOI:** 10.1038/s41598-018-20632-6

**Published:** 2018-02-01

**Authors:** Rebecca L. Ball, Palak Bajaj, Kathryn A. Whitehead

**Affiliations:** 1 Department of Chemical Engineering, Carnegie Mellon University, Pittsburgh, Pennsylvania United States; 2 Department of Biomedical Engineering, Carnegie Mellon University, Pittsburgh, Pennsylvania United States

## Abstract

Oral delivery, a patient-friendly means of drug delivery, is preferred for local administration of intestinal therapeutics. Lipidoid nanoparticles, which have been previously shown to deliver siRNA to intestinal epithelial cells, have potential to treat intestinal disease. It is unknown, however, whether the oral delivery of these particles is possible. To better understand the fate of lipid nanoparticles in the gastrointestinal (GI) tract, we studied delivery under deconstructed stomach and intestinal conditions *in vitro*. Lipid nanoparticles remained potent and stable following exposure to solutions with pH values as low as 1.2. Efficacy decreased following exposure to “fed”, but not “fasting” concentrations of pepsin and bile salts. The presence of mucin on Caco-2 cells also reduced potency, although this effect was mitigated slightly by increasing the percentage of PEG in the lipid nanoparticle. Mouse biodistribution studies indicated that siRNA-loaded nanoparticles were retained in the GI tract for at least 8 hours. Although gene silencing was not initially observed following oral LNP delivery, confocal microscopy confirmed that nanoparticles entered the epithelial cells of the mouse small intestine and colon. Together, these data suggest that orally-delivered LNPs should be protected in the stomach and upper intestine to promote siRNA delivery to intestinal epithelial cells.

## Introduction

RNA interference (RNAi) therapies have the potential to benefit patients suffering from intestinal maladies by reducing or eliminating the production of proteins implicated in intestinal disease^1–4^. These therapies would be most directly delivered by the oral route, which is also the most patient-friendly mode of drug administration. Unfortunately, nucleic acids, such as siRNA, do not survive the harsh environment of the gastrointestinal (GI) tract due to mucus particle trapping and digestion by the abundant enzymes^5^. An ideal delivery vehicle would protect siRNA from the GI environment and enable drug entry into the cytoplasm of intestinal cells, where gene silencing at the mRNA level occurs.

Intestinal diseases such as inflammatory bowel disease and gastrointestinal cancer affect millions of people worldwide and are associated with weight loss, diarrhea, rectal bleeding, and even death^6,7^. Common treatments for inflammatory bowel disease patients include aminosalicylates, corticosteriods, and immunosuppresives^8^. Unfortunately, these drugs are often accompanied by side effects that can include hypertention, osteoporosis, depression, and increased susceptibility to infections^9–11^. These side effects are caused, in large part, by the non-specificity of drug action. One alternative approach that may offer a distinct, more specific mechanism of action with reduced side effects is the use of RNA interference. RNAi therapy could be used to treat intestinal diseases, such as Crohn’s disease or colorectal cancer, that are associated with the upregulation of specific proteins^1,2,4^.

The oral delivery of short interfering RNA (siRNA), the drug that mediates RNA interference, would be the most direct administration route to treat diseased tissue. In addition to avoiding the parenteral circulation, oral delivery would facilitate patient acceptance and reduce complications associated with non-compliance^12,13^. This would offer an improvement over current intestinal therapeutics, which are more invasive and are often administered intravenously or rectally^6,14^.

Several pre-clinical studies have previously reported on oral siRNA delivery using polymeric nanoparticles and microparticles^5,15–19^. For example, oral siRNA particle systems have been used to downregulate TNF-α overproduction in the immune cells located on the basolateral side of the intestinal epithelium, to reduce intestinal and systemic inflammation^15–17,20–23^. Little focus has been placed on siRNA delivery to intestinal epithelial cells, home to several proteins involved in disease progression. Although gene silencing in epithelial cells can be challenging due to their rapid proliferation and shedding^24^, some siRNA delivery systems have overcome this issue. For example, a recent study found that orally delivered hyaluronic acid polymeric nanoparticles embedded in a chitosan/aliginate hydrogel prevented overexpression of an intestinal epithelial and macrophage receptor in an inflammatory bowel disease mouse model^25^. Additionally, nanoparticles-in-microsphere oral system (NiMOS) particles have been shown to deliver siRNA and downregulate cyclin D1, a cell cycle regulatory protein that is overexpressed in colon cancer cells^26,27^.

In previous work, our lab employed lipidoid nanoparticles (LNPs) to deliver siRNA to intestinal epithelial cells for potent and non-cytotoxic gene knockdown^28^. LNPs contain amphiphilic lipid-like molecules, termed lipidoids, which are the active delivery components of the vehicle^29^. We previously found that the lipidoid, 306O_13_, potently silenced genes in a human intestinal cell line, Caco-2. As such, it is used for all experiments in this study. LNPs were formulated by adding the lipidoid to a mixture of cholesterol, DSPC, and PEG-lipid, which improve nanoparticle stability and efficacy. The lipid mixture was then mixed with an aqueous siRNA solution, which prompted nanoprecipitation due to hydrophobic and electrostatic driving forces. LNPs have been shown to induce gene knockdown *in vivo* without causing detectable toxicity or immunogenicity upon systemic administration of siRNA doses up to 3 mg/kg^30^. Because they are broadly effective, we would like to investigate the robustness and biodistribution of LNPs in the GI tract, with an ultimate goal of oral LNP delivery. However, the environment of the GI tract has proven to be a stability challenge for countless drugs and delivery vehicles. Herein, we evaluate LNP fate and function in the GI tract to improve our understanding of LNP potential as an oral siRNA delivery vehicle.

## Results

### LNPs maintain potency in a wide range of pH solutions

The pH of the GI tract ranges from 1–2 in the stomach to 8 in the large intestine^31^. A concern for oral siRNA LNP delivery is that LNPs may not be stable under such a broad range of pH conditions. Therefore, LNP stability as a function of pH was investigated by formulating the LNPs in PBS at a pH of 7.4 and then diluting them into PBS buffered to pH values of 1–9. After 30 minutes of incubation, LNPs were characterized and evaluated for their ability to induce gene silencing. Gene silencing was assessed by transfecting HeLa cells that stably express firefly and *Renilla* luciferase with LNPs containing siRNA against the firefly luciferase gene. *Renilla* luciferase served as a negative control, and reductions in *Renilla* luciferase would be suggestive of cytotoxicity or off-target effects.

**Figure 1:**
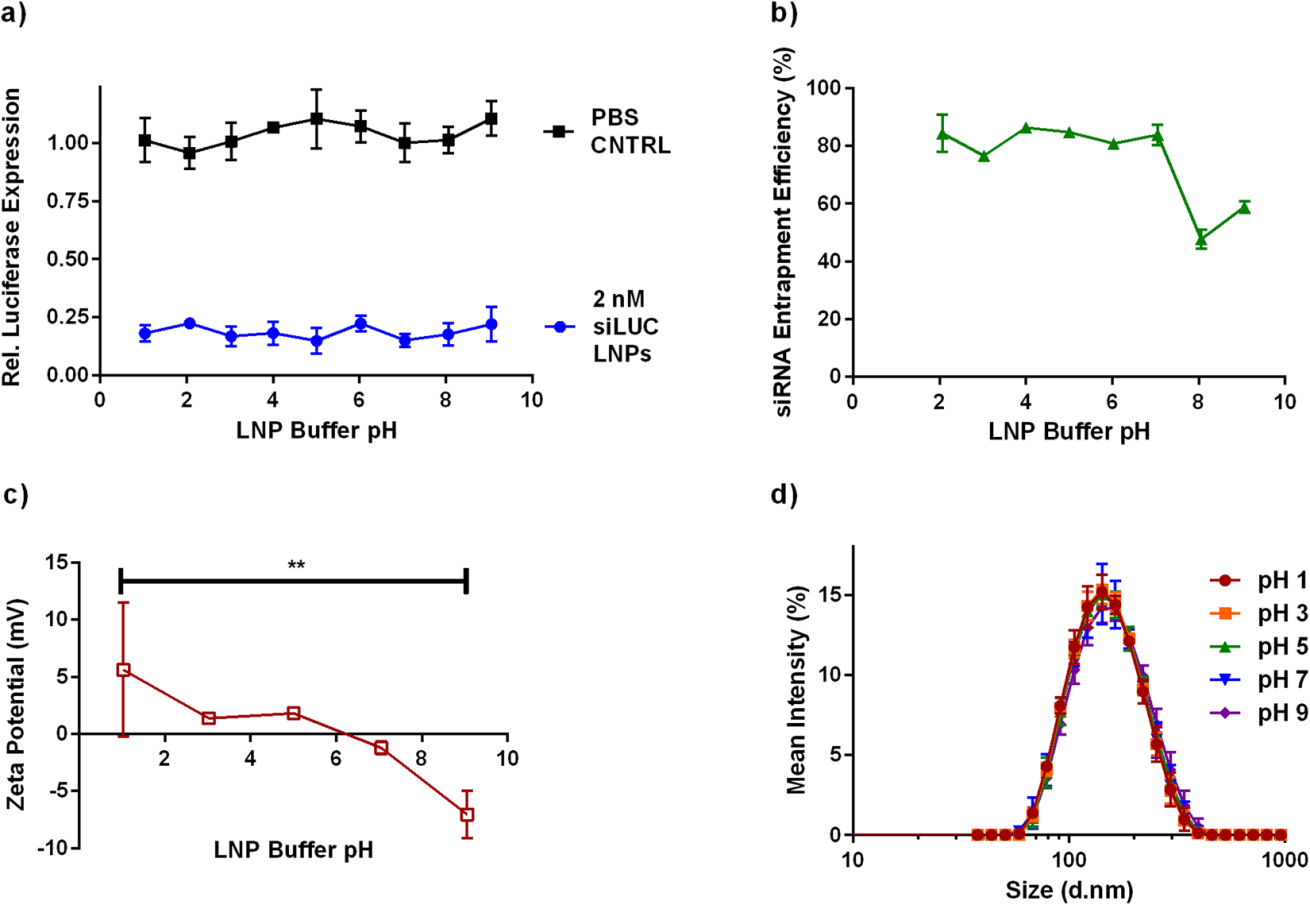
The pH of lipid nanoparticle (LNP) buffer does not affect gene silencing. LNPs were formulated and then diluted into phosphate buffered saline (PBS) that had been titrated to a final pH of 1–9. (**a**) Gene silencing induced by a 2 nM dose of LNP-encapsulated siRNA did not vary with LNP buffer pH (blue circles). Buffer alone had no effect on luciferase expression (black squares). (n = 4) (**b**) siRNA entrapment was constant over a pH range of 2–7. (**c**) LNP charge was a function of buffer pH, with LNPs becoming more positive at lower pH and more negative at higher pH. A one-way ANOVA indicated significance. **p = 0.0015 (**d**) LNP buffer pH had minimal effect on nanoparticle size and PDI. Dynamic light scattering (DLS) was used to measure the size and polydispersity (PDI) of the LNPs in titrated PBS. (n = 3 technical replicates for panels **b**–**d**). In all panels, error bars represent s.d.

As seen in Fig. 1a, LNPs induced ~80% luciferase gene silencing in HeLa cells at an siRNA dose of 2 nM, regardless of buffer pH (blue circles). Controls were conducted in which HeLa cells received the same volume of titrated PBS as the transfected samples. Since changes in buffer pH did not significantly alter luciferase gene expression (black squares), we concluded that LNP efficacy is not a function of pH. LNP siRNA entrapment efficiency, shown in Fig. 1b, remained around 85% for all pH values up to 8, at which point entrapment dropped to ~50%. Interestingly, this decrease in measured siRNA entrapment did not correlate with reduced efficacy. Similarly, as seen in Fig. 1d, buffer pHs between 1 to 7 did not affect the z-average diameter or polydispersity index (PDI) of the LNPs, with values of approximately 140 nm and 0.12, respectively. LNPs incubated in pH 9 buffer showed a slight increase in size and PDI at 150 nm and 0.17, respectively. As expected, surface charge, measured by zeta potential, varied with buffer pH. The LNP surface charge became slightly positive in lower pH PBS and slightly negative at pHs of 7–9 (Fig. 1c). The change in zeta potential can be attributed to protonation of lipidoid amines under low pH conditions. On the whole, these experiments suggested that the changes in pH that LNPs would experience as they move through the GI tract will not adversely affect their functionality.

### Pepsin and bile salts reduce LNP efficacy

In addition to drastic pH changes, the GI tract contains digestive enzymes that break down proteins, nucleic acids, sugars and other nutrients during digestion. Therefore, we next asked whether or not these enzymes impact LNP delivery efficacy. We tested LNPs after a simulated *in vitro* digestion in which they were exposed to the enzymes pepsin and pancreatin, as well as bile salts. Pepsin is found in the stomach and degrades proteins. Pancreatin, a secretion of the pancreas, is a mixture of enzymes that include trypsin, amylase, lipase, ribonuclease, and proteases^32^. Along with bile salts, which emulsify lipids, pancreatin and pepsin digest macromolecules into their building blocks for absorption or clearance from the body^33–35^.

**Figure 2:**
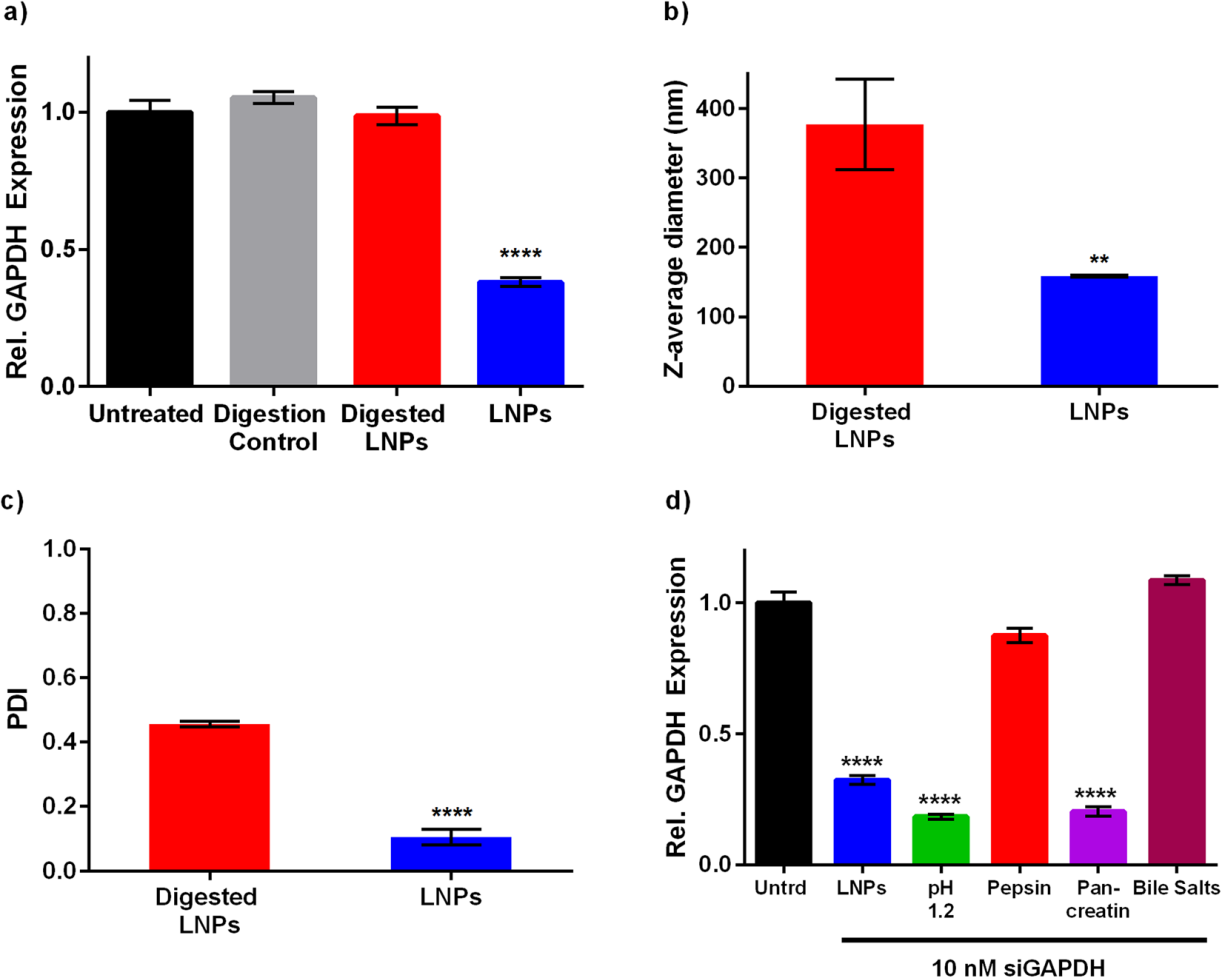
Simulated digestion media inhibits LNP delivery activity. (**a**) At a dose of 10 nM, LNPs loaded with siRNA against GAPDH induced ~70% GAPDH knockdown (blue bar). Efficacy was completely inhibited when LNPs were incubated in digestion media prior to transfection (red bar). (**b**) The z-average diameter and (**c**) PDI of LNPs increased after incubation in digestion media. **p = 0.0045 and ****p < 0.0001 vs. digested LNPs (n = 3). (**d**) When LNPs were subject to “deconstructed” digestion media, only pepsin and bile salts inhibited LNP siRNA delivery efficacy. ****p < 0.0001 vs. untreated. (n = 3). Error bars in all panels represent s.d.

LNP “digestion” was performed in two steps. First, formulated LNPs were incubated in simulated gastric fluid that contained pepsin (pH 1–2). After 30 minutes, they were exposed to a simulated intestinal fluid containing pancreatin and incubated for 30 minutes. LNPs were then delivered to Caco-2 cells, which are an established *in vitro* model of intestinal epithelial cells^36,37^. LNPs contained siRNA specific against the housekeeping gene, GAPDH, which was dosed at 10 nM total siRNA. Gene silencing was assessed 24 hours later by quantitative PCR. As seen in Fig. 2a, the digested LNPs were ineffective, while the undigested LNPs achieved ~70% gene silencing. Potency may have been reduced by aggregation of the LNPs, as seen in the significant increase in the z-average diameter and PDI of the digested LNPs compared to the original nanoparticles (Fig. 2b,c). To identify the cause of the efficacy reduction, we deconstructed the *in vitro* digestion process. LNPs were examined after exposure to either pH 1.2 buffer, pepsin, pancreatin, or bile salts at concentrations representative of “fed” conditions in humans. Figure 2d shows that pepsin and bile salts both negatively impacted LNP efficacy.

**Figure 3:**
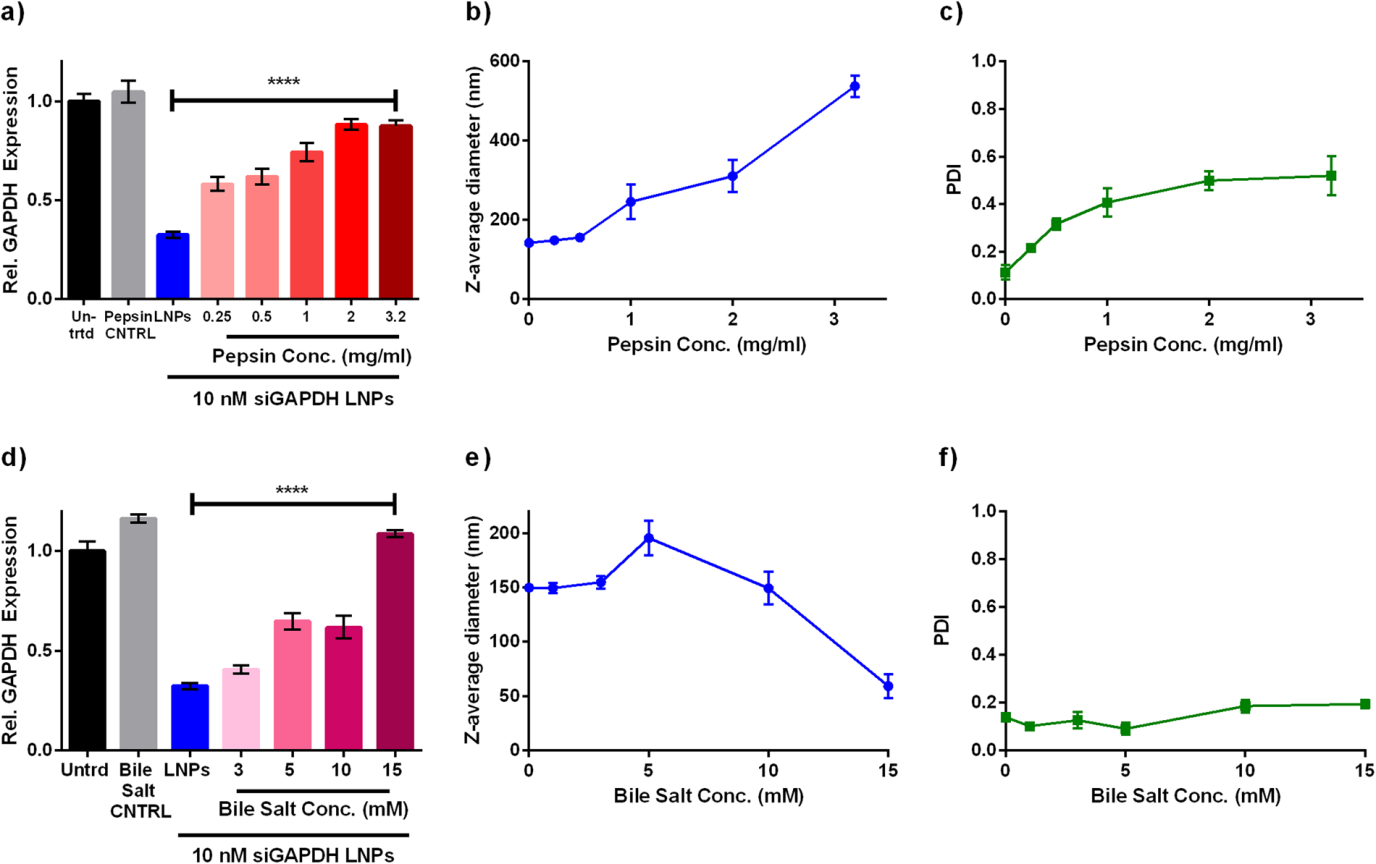
Pepsin and bile salts inhibit gene silencing in a dose-dependent manner. After formulation, LNPs were diluted in pepsin or bile salt solutions and then incubated at 37 °C for 30 minutes. (**a**) LNPs silenced GAPDH less effectively (siRNA dose = 10 nM) with increasing pepsin concentration. Pepsin alone did not affect GAPDH expression (gray bar). A one-way ANOVA indicated significance. ****p < 0.0001 (n = 3) (**b**) The size and (**c**) PDI of LNPs exposed to pepsin solutions increased with increasing pepsin concentration. (n = 3 technical replicates) (**d**) Bile salt concentration was inversely proportional to LNP efficacy (siGAPDH dose = 10 nM). A concentration of 3 mM, representative of the fasting state, did not reduce efficacy. Bile salts alone did not affect GAPDH expression (gray bar). A one-way ANOVA indicated significance. ****p < 0.0001 (n = 3) (**e**) The size of LNPs decreased at a bile salt concentration of 15 mM. (**f**) The PDI of LNPs was not a function of bile salt concentration. (n = 3 technical replicates). Error bars in all panels represent s.d.

Subsequently, we asked how pepsin and bile salt concentration influenced gene silencing, because digestion enzyme concentration can vary with the timing and nutritional content of meals. The concentrations applied to LNPs in Fig. 2d are representative of higher, fed state concentration of pepsin and bile salts. In this experiment, LNPs loaded with siGAPDH were mixed with increasing concentrations of pepsin (0–3.2 mg/mL) and delivered to Caco-2 cells at an siRNA dose of 10 nM. Although LNP efficacy declined with increasing pepsin concentration (Fig. 3a), potency was partially retained at “fasting” pepsin concentrations (0.25 mg/mL). LNP diameter and PDI increased with pepsin concentration (Fig. 3b,c) while the DLS particle count rate remained constant, suggesting that the enzyme caused LNP aggregation which prevented cell uptake and/or efficient gene silencing in Caco-2 cells.

A similar trend was seen when the LNPs were mixed with increasing concentrations of bile salts (3–15 mM) (Fig. 3d). LNPs exposed to 3–10 mM (fasting concentrations) of bile salts retained most of their gene silencing ability in Caco-2 cells. However, the 15 mM bile salt concentration completely prevented gene silencing. LNPs incubated with bile salts maintained their original size at bile salt concentrations up to 10 mM (Fig. 3e). The PDI (Fig. 3f) and the particle count rate (data not shown) did not change with concentration. Together, these data indicate that oral LNP delivery should be conducted under fasting conditions to facilitate LNP stability and retain the majority of LNP gene silencing ability.

### Mucin prevents LNP gene silencing *in vitro*

**Figure 4:**
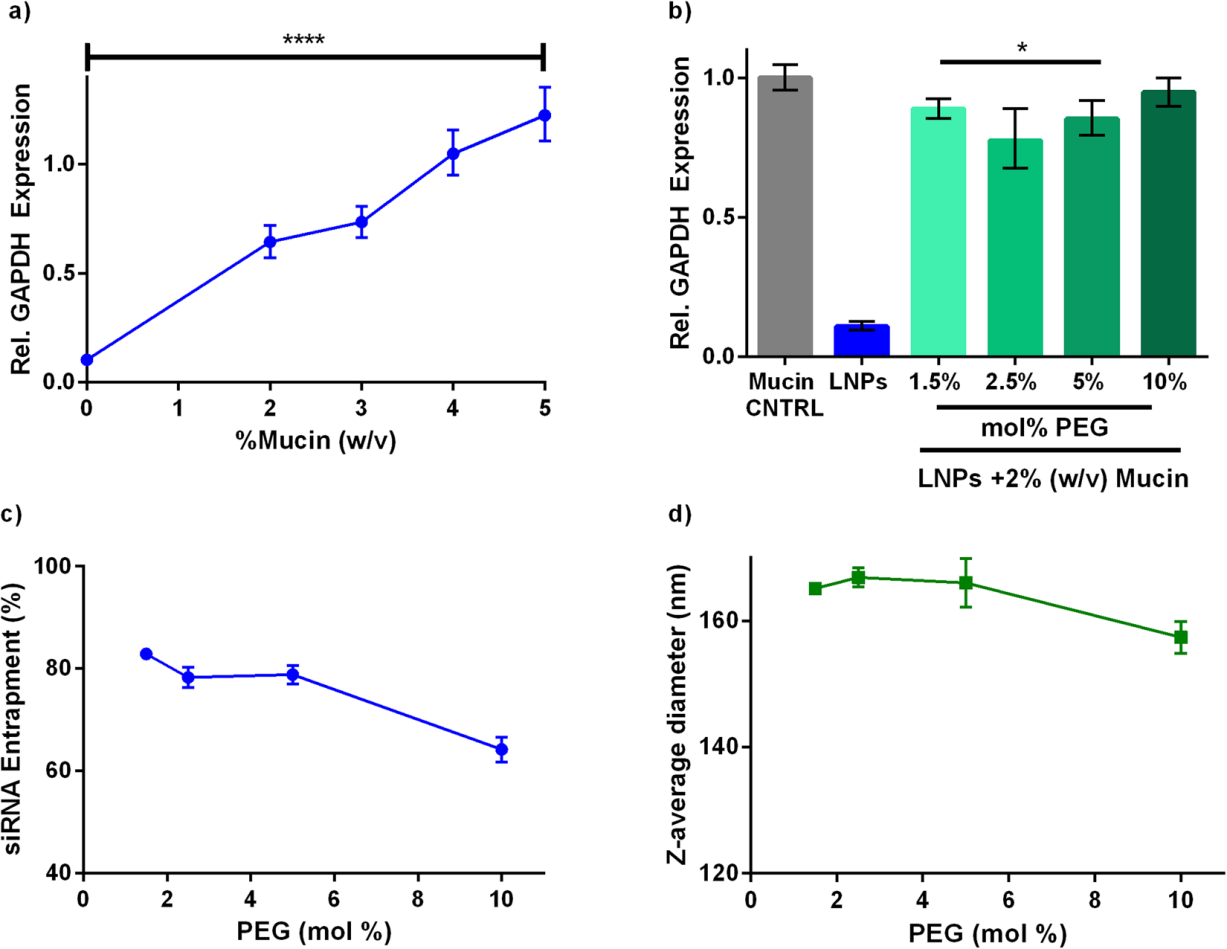
Mucin inhibits LNP-mediated gene silencing. Caco-2 cells were cultured with mucin, and LNPs were pipetted on top of the mucin layer. (**a**) Mucin inhibited LNP-mediated gene silencing (siGAPDH dose = 100 nM) as a function of concentration. Each data point was normalized to GAPDH expression for control wells receiving mucin only. A one-way ANOVA was performed. ****p < 0.0001 (n = 3) (**b**) When 2% (w/v) mucin was present, LNPs formulated with 2.5 mol% PEG induced the most gene silencing. *p = 0.0268 (1.5%), p = 0.0228 (2.5%), p = 0.0316 (5%) vs. mucin control. (**c**) siRNA entrapment and (**d**) LNP diameter decreased with increasing PEG mol% in the LNP formulation. (n = 3). Error bars in all panels represent s.d.

Mucus, which coats the intestinal epithelium, serves as a physical barrier that hinders the transport of macromolecules. While this physiological barrier prevents bacteria and other toxins from accessing epithelial tissue, it also inhibits the delivery of lipid nanoparticles to their cellular targets. The mucus barrier of the intestines is 200–800 µm thick and has a short turnover time of several hours that facilitates the rapid clearance of undigested compounds^38,39^. To test LNP diffusion through mucus, siGAPDH-loaded LNPs were delivered to Caco-2 cells that were incubating in culture media containing 2–5% (w/v) mucin type II. These mucin concentrations are representative of those observed in human intestines^40^. As seen in Fig. 4a, mucin affected LNP gene silencing in a concentration dependent manner. Even low concentrations of mucin (2% w/v) significantly reduced gene silencing from ~90% to ~40% at an siRNA dose of 100 nM. These experiments suggest that LNPs do not readily diffuse through mucin to enter the Caco-2 cells.

Previous studies have reported that an increase in PEG density on the outside of a particle can improve diffusion through mucus^41^. Therefore, we asked whether increasing the PEG mol% in the LNP formulation above the standard 1.5 mol% would improve gene silencing in the presence of mucin. LNPs were formulated with 1.5–10 mol% PEG and pipetted on top of the mucin-covered Caco-2 cells. Twenty-four hours post-transfection, nanoparticles containing 2.5 mol% PEG resulted in the most gene silencing, as seen in Fig. 4b. Although this value was not significant compared to 1.5 and 5 mol% PEG, there is likely a trade-off between improving diffusion and inhibiting target cell uptake^42,43^. LNPs formulated with higher mol% PEG had lower siRNA entrapments and z-average diameters (Fig. 4c,d).

### LNPs do not affect tight junction integrity

**Figure 5:**
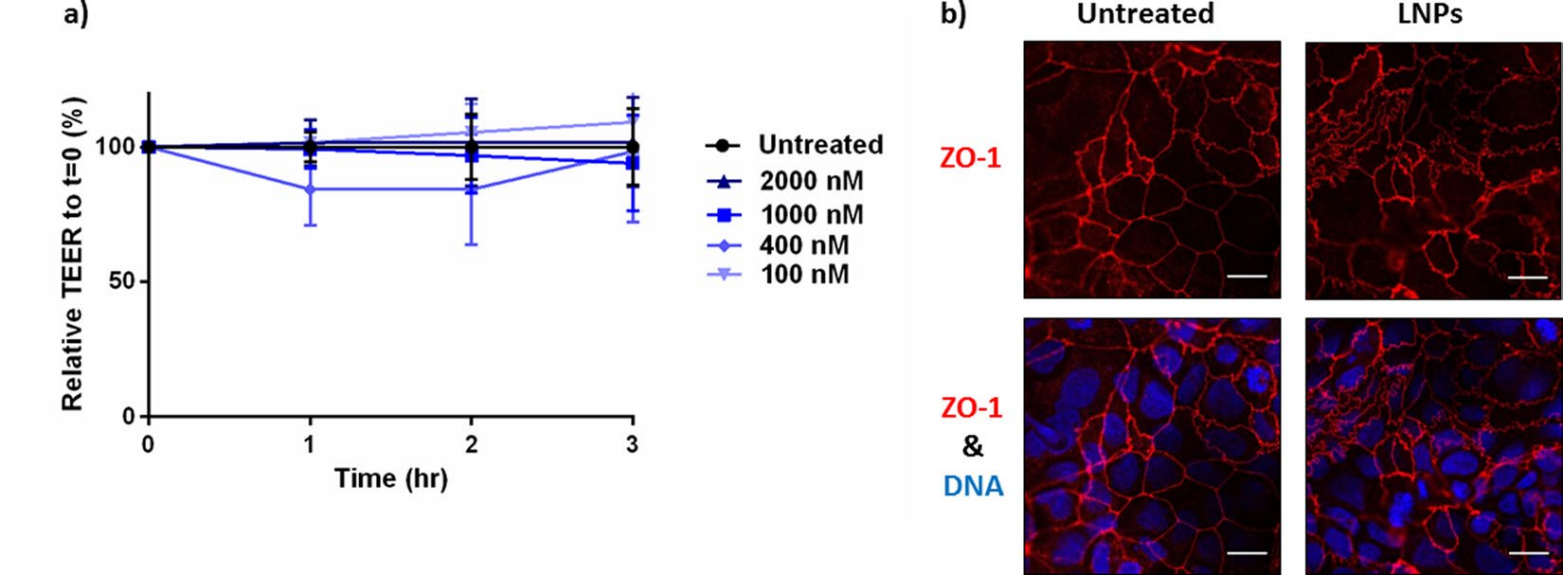
LNPs did not adversely affect Caco-2 intestinal permeability or ZO-1 arrangement. TEER values are relative to t = 0 at the time of LNP transfection and normalized to untreated cells. (**a**) LNPs, even at very high doses, do not alter TEER measurements in Caco-2 monolayers over a period of 3 hours. TEER values are relative to initial TEER measurements. Error bars represent s.d. (n = 3) (**b**) LNPs do not affect ZO-1 tight junction arrangement. Caco-2 monolayers were fixed and stained three hours after siRNA LNP delivery. ZO-1 and Hoechst stains appear in red and blue, respectively. Scale bars = 15 µm.

We also evaluated LNPs for their effect on intestinal epithelial tight junctions. Tight junctions are protein complexes that form between intestinal cells that restrict transport of macromolecules from the apical to the basolateral side of the intestinal epithelium^44^. In several intestinal diseases, including inflammatory bowel disease and colon cancer, persistent inflammation results in the loosening of tight junction, adversely affecting the integrity of the intestinal barrier. To avoid further exacerbation of this condition, it is important that intestinal therapeutics do not affect tight junctions. To determine LNP influence on tight junction integrity, we cultured Caco-2 cells into monolayers that recapitulate intestinal cell arrangement and tight junction formulation *in vivo*. One to three hours after delivery of LNPs to Caco-2 monolayers, tight junction integrity was assessed by transepithelial electrical resistance (TEER). Reductions in resistance would be indicative of loosening of tight junctions and decrease in barrier function. As seen in Fig. 5a, there was no significant change in TEER, even at very high LNP-siRNA doses, suggesting that LNPs do not affect barrier function. After three hours, some of the monolayers were fixed and stained to visualize the arrangement of the tight junction protein, zonula occludens-1 (ZO-1). The localization of ZO-1 did not change in the presence of 100 nM siRNA LNPs (Fig. 5b).

### LNPs remain in the mouse GI tract for at least 8 hours post-delivery

**Figure 6:**
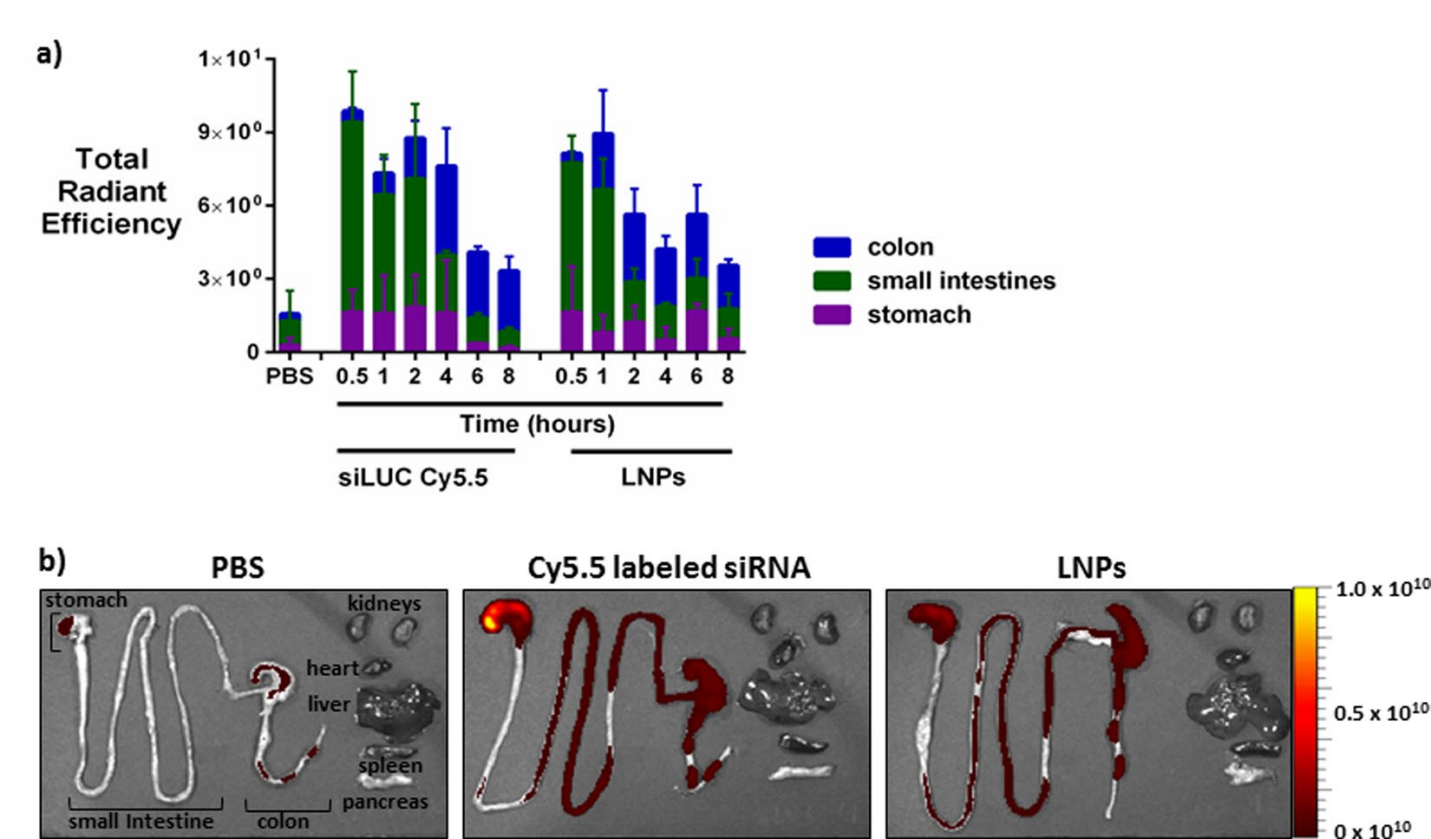
LNPs remained in the GI tract for at least 8 hours after oral gavage in mice. Female C57BL/6 mice were fasted for 4 hours and orally gavaged with buffer (PBS), Cy5.5-labeled siRNA, or LNPs with Cy5.5 labeled siRNA. Total siRNA dose was 0.5 mg/kg. (**a**) The fluorescence signal after oral LNP delivery persisted for at least 8 hours in the GI tract of mice. Most of the signal was contained within the small intestine. Error bars represent s.d. (n = 3–4). (**b**) Sample images depict siRNA location 4 hours post-gavage. Total radiant efficiency units: [p/s]/[µW/cm²]. Color scale: Min = 1.04e8 Max = 1.00e10.

We have previously shown that LNPs enter Caco-2 intestinal cells and elicit potent gene silencing in these cells *in vitro*^28^. The next question is, what will happen to the nanoparticles after oral delivery *in vivo*? First, we studied LNP biodistribution after a single oral dose of LNPs containing Cy5.5-labeled siRNA in C57BL/6 mice. We also conducted PBS and naked Cy5.5-labeled siRNA controls. The mice were sacrificed 30 minutes to 8 hours after the oral dose, and their excised organs were imaged for fluorescent signal using an *in vivo* imaging system (IVIS). Figure 6a shows this quantified signal as a function of time for the stomach, small intestine, and colon. Both naked siRNA and LNPs moved through the stomach and into the small intestine by 30 minutes post-administration. Over the next several hours, fluorescent signal increased in the colon as the samples moved through the GI tract. Total signal decreased over time as siRNA was eliminated from the GI tract. Figure 6b shows representative fluorescent images at 4 hours post-gavage. There was no significant siRNA signal in the kidneys, heart, liver, spleen, or pancreas, suggesting that a negligible amount of naked siRNA or the LNPs crossed the epithelial barrier to enter the blood stream.

**Figure 7:**
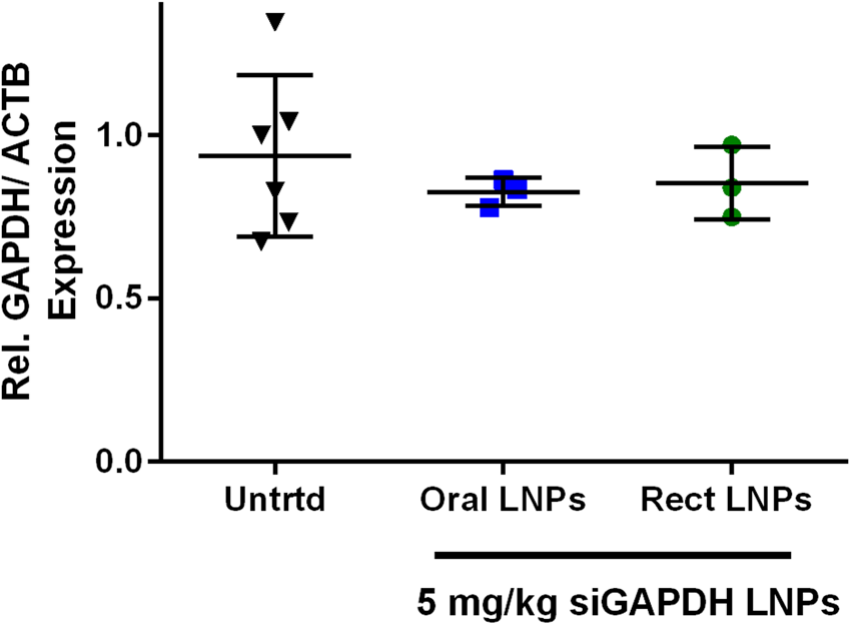
LNPs did not significantly silence GAPDH in the mouse colon. Mice received either an oral gavage or rectal administration of LNPs at an siGAPDH dose of 5 mg/kg. Mice were sacrificed 24 hours later. The colons were first washed with PBS and the intestinal mucosa was scraped off in the rectal area for mRNA extraction and qPCR. (n = 3).

After confirming LNP biodistribution after oral gavage in mice, we tested gene silencing efficacy in mouse colon cells following a single oral or rectal dose of LNPs. In these experiments, LNPs loaded with siGAPDH were delivered by oral gavage or rectal administration to mice at an siRNA dose of 5 mg/kg. Viewing Fig. 7, the average GAPDH gene expression was lower in mice that received LNPs; however, average expression was not statistically significant from expression in the untreated mice GAPDH levels. Obtaining statistical significance was hindered, at least in part, by the substantial variability in GAPDH expression levels in untreated mouse colon cell samples. Based on our mucin data, we suspect that an insufficient number of LNPs are able to diffuse to the vicinity of the intestinal epithelial cells to significantly knockdown gene expression.

### LNPs enter the cells of the small intestine and colon

**Figure 8:**
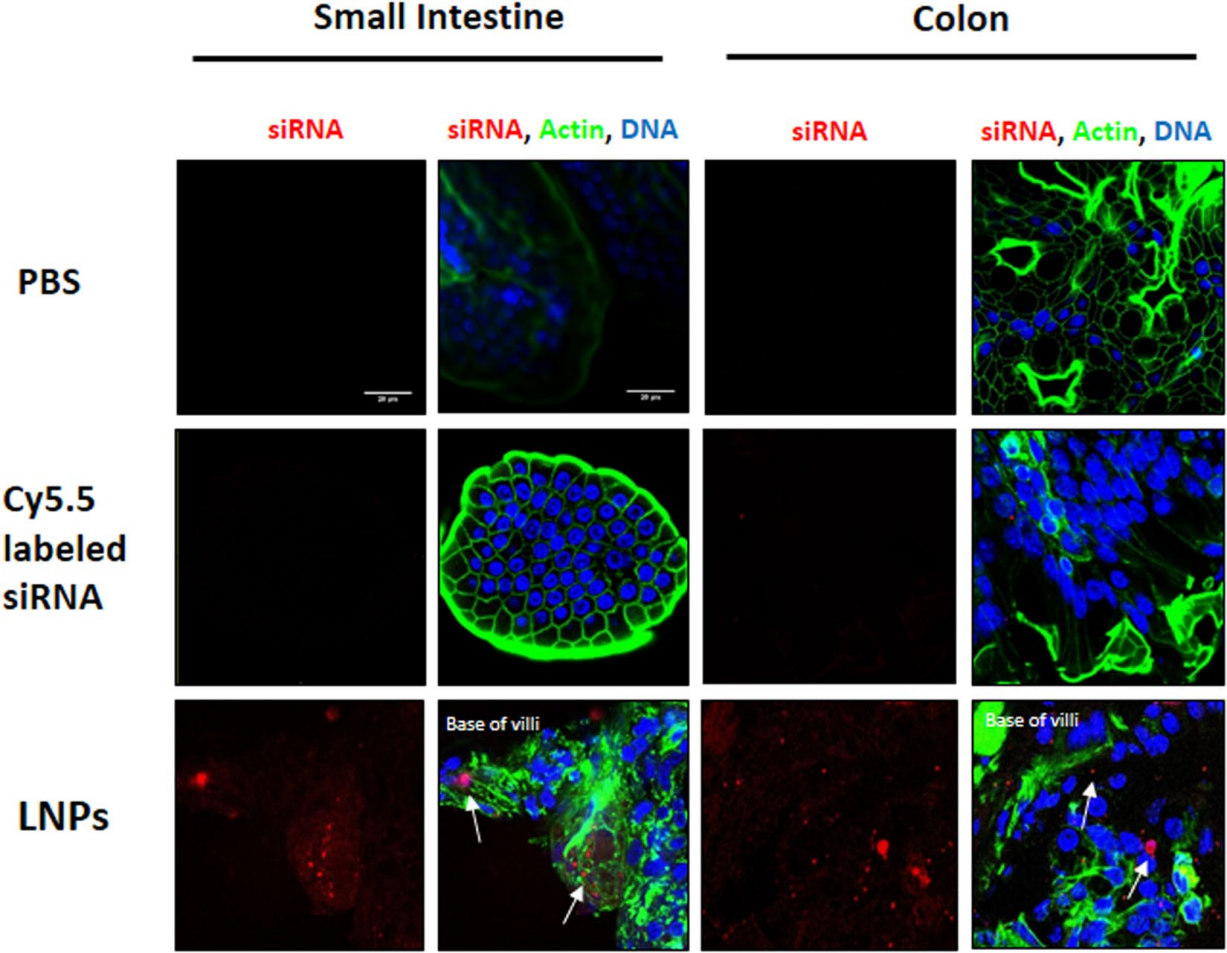
The LNPs were visualized in the small intestine and colon epithelial cells of mice. Mice received an oral gavage of either naked or LNP-encapsulated Cy5.5-labeled siRNA (0.5 mg/kg). Mice were sacrificed after 6 hours and tissue sections were fixed and stained for DNA (blue) and actin (green). siRNA appears in red. Scale bars are 20 µm (magnification = 63×).

To investigate the lack of gene silencing further, confocal microscopy was used to visualize intestinal cell entry in mice. Tissue sections from mice that received an oral gavage of LNPs loaded with Cy5.5-labeled siRNA were washed, fixed and stained for DNA and actin. Control mice received either PBS or naked Cy5.5-labeled siRNA. As shown in Fig. 8, only the mice that received LNP-formulated siRNA had significant Cy5.5 fluorescence signal in the small intestinal and colon epithelial cells. Surprisingly, some LNPs penetrated to the base of the villi in the small intestine and colon, indicating that at least a subset of LNPs successfully diffuse to the intestinal tissue. LNP uptake across samples and within the samples shown was not consistent, however, and it is likely that a greater percentage of intestinal cells must receive LNPs to result in gene silencing.

## Discussion

RNAi therapeutics have the potential to offer a mechanistically distinct form of therapy for several intestinal diseases, including inflammatory bowel disease, colon cancer, and irritable bowel syndrome^2,4,45^. Unfortunately, the oral delivery of nucleic acids is challenging due to the harsh environment of the GI tract^14^. Specifically, nucleic acids like siRNA must survive low pH conditions, avoid nucleases, and penetrate the mucosal barrier to reach the target intestinal epithelial cells^14^. Most siRNA delivery systems, including lipidoid nanoparticles, were developed with systemic delivery in mind. Understanding their stability and transport properties in the GI tract is important if their use is to be extended for oral delivery and the treatment of intestinal disorders.

Transit through the GI tract exposes a therapeutic to a wide range of pH values, from 1–2 in the stomach to approximately 8 in the large intestine^31^. Our experiments (Fig. 1) demonstrated that LNPs maintained stability and gene silencing efficacy in HeLa cells following exposure to a wide range of buffer pHs (1–9). Similar results had been found previously for long term LNP storage in solutions of different pH^46^. These data suggest that pH instability in the GI tract will not adversely affect lipid nanoparticle mediated gene silencing.

Digestive enzymes in the GI tract may pose a greater challenge. We selected enzyme concentrations based on human studies. The fed concentration of pepsin has been found to range from 0.26–1.72 mg/mL, and the fasted concentrations can range from 0.11–0.87 mg/mL^34^. The wide range in reported literature values is due to differences in experimental methods for measuring pepsin concentrations in humans.

Unfortunately, pepsin at high physiological concentrations prevented LNP gene silencing in Caco-2 cells. Based on the data in Fig. 3a, LNPs would need to be administered to the patient under fasting conditions to minimize the aggregation effect of pepsin. A similar study investigating nanoparticle stability in the presence of pepsin reported that solid lipid nanoparticles, nanostructured lipid carriers, and lipid nanoemulsions did not change particle size in the presence of pepsin at a concentration representative of a fasted stomach (0.32% w/v)^47^. However, at the same pepsin concentration, a study of miltefosine-loaded lipid nanoparticles reported a three-fold increase in particle size^48^. The mechanism of pepsin-induced LNP aggregation is unclear. Previous studies have reported that PEG and pepsin may interact electrostatically through the protonated carboxyl groups of the pepsin and the ether of the PEG by hydrogen bond formation^49^. Spelzini and colleagues observed that lower molecular weight PEGs, like the one used on LNPs, are more hydrophobic in an extended conformation with a higher capacity to interact with the pepsin protein domain^50^. The pepsin and PEG2000 on the outer surface of the LNPs could be favorably interacting to cause the formation of aggregates that prevent the LNPs from entering the cell.

Bile salts, another class of molecule that aids digestion, also prevented gene silencing at high concentrations. In the small intestines of humans, the fasted bile salt concentration is reported to be 2.6–6.4 mM and the fed concentration is 11–14.5 mM^33,34,51^. There is a possibility the LNPs would still result in potent intestinal gene silencing when the patient has fasted beforehand. Interestingly, the z-average diameter of the LNPs drastically decreased at a bile salt concentration of 15 mM. It is unclear why the high concentration reduced LNP size, however, it could be due to an interaction between the lipidoids in the LNP and bile salts. Bile salts are amphiphilic and self-assemble into micelles in an aqueous environment^33^. It is possible that at high concentrations, bile salts (15 mM) insert themselves into the lipid bilayer of the LNPs or solubilize some of the lipophilic components, leading to a change in particle formation. The size reduction could lead to less siRNA entrapment within the particles and therefore a loss of gene silencing efficacy.

The presence of mucin in Caco-2 cell culture also inhibited LNP gene silencing in a concentration dependent manner. The size, surface chemistry, and charge of LNPs play likely roles in their interactions with mucus. Another class of nanoparticles known as mucus penetrating particles (MPPs) have been shown to diffuse through mucus at speeds comparable to diffusion in water. These MPPs are ~100 nm in diameter, neutrally charged, and have a high density of low molecular weight PEG on the surface^38,52^. LNPs have similar structures to MPPs, except LNPs have much lower PEG surface density. In addition to aiding in mucus diffusion, nanoparticle PEGylation can also reduce non-specific protein aggregation, and immune cell uptake that hinders efficacy^53–56^. Unfortunately, we found that a large increase in PEG mol% in the LNPs reduced efficacy, likely because of shielding effects and decrease in siRNA entrapment. It is not clear if there is a way to formulate LNPs to facilitate their ready diffusion through mucus while retaining efficacy.

Fortunately, even though pepsin, bile salts, and mucin reduced gene silencing *in vitro*, the LNPs entered mouse small intestine and colon cells after oral delivery. The fluorescent siRNA signal penetrated down to the base of the intestinal villi, close to the lamina propria area of the mouse intestines. It is possible LNPs became more concentrated in certain areas of the mouse intestines than others, and this led to diffusion into the intestinal crypts because of a larger concentration gradient. This could be beneficial for delivering siRNA to immune cells that are present in the area. Unfortunately, even though signal from LNPs with siRNA was seen within small intestine and colon cells of mice, there was no significant gene silencing attained after oral or rectal delivery. There is a possibility the dose delivered (5 mg/kg) isn’t sufficient to achieve full coverage of LNPs over the majority of epithelial cells. Furthermore, when excising intestinal sections for mRNA extraction, the small tissue sample that is taken may not be representative of the remaining length of the intestine. In other words, it is possible that there are localized sections of intestine where epithelial silencing is occurring. We found that different sections of the colon experienced varying degrees of gene silencing with no apparent pattern. Uniform delivery throughout the intestines may be the greatest remaining challenge to achieving siRNA-mediated gene modulation in the intestinal epithelium.

Based on this work, it is recommended that the particles be protected, at the very least, in the stomach to prevent pepsin inactivation. One option is particle encapsulation with a pH responsive polymer (e.g. Eudragit®) that will not dissolve until a specific pH is reached within the GI tract^57,58^. Another option would involve loading lyophilized LNPs^46^ into a capsule coated with a pH responsive polymer. Alternatively, it may be possible to decorate LNPs with a high density of responsive polymer chains that provide steric hindrance to enzymatic degradation and are programmed to be released prior to cell entry.

## Methods

### Materials

Cholesterol was purchased from Sigma Aldrich, while distearoyl-sn-glycerol-3-phosphocholine (DSPC) and PEG2000-DMG were obtained from Avanti Polar Lipids. Dual HeLa and Caco-2 cells were purchased from American Type Culture Collection (Manassas, VA). Porcine pepsin, pancreatin, bile salts, and mucin type II were obtained from Sigma (St Louis, MO). Dulbecco’s Modified Eagles Media (DMEM), trypsin, penicillin/streptomyocin, phosphate buffered saline (PBS), fetal bovine serum (FBS), and GAPDH siRNA were purchased from Thermo Fisher (Waltham, MA). Corning BioCoat HTS 1 µm porous support Transwell plates, basal seeding medium (BSM), Corning EnteroSTIM enterocyte differentiation medium (EDM), and MITO + serum extender were obtained from VWR (Radnor, PA). Anti-firefly luciferase siRNA (siLuc) was purchased from Dharmacon (Lafayette, CO).

### LNP formulation

LNPs were prepared as previously described^30^. A lipid solution was prepared by dissolving the 306O_13_ lipidoid^28^, cholesterol, DSPC, and PEG2000-DMG in ethanol and mixed at a molar ratio of 50:38.5:10:1.5 in a solution of 90% ethanol and 10% 10 nM sodium citrate (by volume). An siRNA solution was prepared by diluting siRNA in 10 nM sodium citrate such that the final weight ratio of lipidoid: siRNA was 5:1. Particles formed upon brief vortexing of equal volumes of siRNA solution with the lipid solution. The LNPs were diluted in phosphate buffered saline (PBS).

### Nanoparticle characterization

The LNPs were diluted in PBS to an siRNA concentration of 1 µg/mL for all characterization studies. In order to measure LNP siRNA entrapment, a Quanti-iT^TM^ Ribogreen^®^ assay (Invitrogen) was used following the manufacturer’s protocol. The particle size and zeta potential of the LNPs was measured using a Malvern Zetasizer Nano (Malvern Instruments, UK) performing three technical replicate runs.

### Cell culture

HeLa and Caco-2 cells were grown in DMEM supplemented with 100 ml/L of FBS, 10 IU/mL of penicillin, and 10 mg/mL streptomyocin. The cells were incubated at 37 °C in a 5% CO_2_ environment and subcultured by partial digestion with 0.25% trypsin and ethylenediaminetetraacetic acid (EDTA). HeLa cell passages 10–50 and Caco-2 cells passages 30–60 were used for the experiments.

### LNP stability in a range of pH solutions

The LNPs were formulated as described above at an anti-luciferase siRNA concentration of 0.125 mg/mL. The LNPs were diluted into PBS at a pH of 1.03, 2.07, 3.03, 4.00, 5.01, 6.03, 7.05, 8.06 or 9.06 to an siRNA concentration of 2.5 µg/mL. The pH of the PBS was altered using 1 M NaOH or 1 M HCl. The LNPs were left at room temperature for approximately 30 minutes and then delivered to Dual HeLa cells at an siRNA concentration of 2 nM for 24 hours. The Dual HeLa cells stably express firefly and *Renilla* luciferase and were seeded at a density of 15,000 cells per well in 96 well plates. Luciferase activity was assessed with a Dual-Glo Luciferase Assay Kit (Promega, Madison, WI) according to the manufacture’s protocol. While firefly luciferase served as the target gene for knockdown, the *Renilla* luciferase activity served as a control.

### *LNP in vitro* GI tract digestion

LNPs were formulated at 0.125 mg/mL siRNA against GAPDH and then diluted to 6.25 ug/mL siRNA into simulated gastric fluid. This fluid contained PBS at a pH of 1.2 with 3.2 mg/mL of pepsin and 0.03 M NaCl. The solution was then incubated at 37 °C for 30 minutes at 100 rpm. For the simulated intestinal fluid, 0.05 M NaHCO_3_ and 4 mg/mL of pancreatin were added to the LNP gastric fluid and the pH was raised to 6 with 1 M NaOH. The solution was again incubated at 37 °C for 30 minutes at 100 rpm. For the broken down GI tract digestion, the LNPs were either incubated at pH 1.2, with pepsin (3.2 mg/mL), pancreatin (4 mg/mL), or bile salts (15 mM) at 37 °C for 30 minutes at 100 rpm. The digested LNPs were delivered to Caco-2 cells seeded the night before at a density of 500,000 cells/well in 6 well plates. An siRNA dose of 10 nM was delivered to the Caco-2 cells for 24 hours. Digestion media containing simulated gastric and intestinal fluids without LNPs were delivered to cells to ensure the digestion media did not affect gene expression.

### LNP stability with pepsin and bile salts

For pepsin stability studies, LNPs were formulated with siRNA against GAPDH at a concentration of 0.125 mg/mL and diluted 10× into a PBS solution at 1.2 pH with 0, 0.25, 0.5, 1, 2, or 3.2 mg/mL porcine pepsin. In the bile salt experiments, the LNPs were formulated at the same siGAPDH concentration and diluted into PBS at a pH of 6 with 3, 5, 10, or 15 mM bile salts. For both stability studies, the LNPs were incubated at 37 °C for 30 minutes on a shaker at 100 rpm. For gene expression experiments, the “digested” LNPs were delivered to Caco-2 cells (density: 500,000 cells/well) at an siGAPDH dose of 10 nM for 24 hours.

### LNPs silencing with Mucin

Caco-2 cells were seeded at a density of 250,000/well in 24 well plates. Porcine gastric mucin type II (Sigma) was dissolved in Caco-2 culture media at 37 °C at a range of 2–5% (w/v). 500 uL of the mucin/culture media solution was pipetted on top of the Caco-2 cells. LNPs formulated with varying mol% of PEG (1.5–10) were delivered on top of the mucin to the Caco-2 cells for 24 hours at a dose of 100 nM.

### RNA isolation, reverse transcription, and quantification of gene expression with qPCR

For gene expression studies, the whole RNA content was isolated from Caco-2 cells using a Qiagen RNeasy Kit according to the manufacturer’s protocol. The total RNA concentration was determined using a Nanodrop 2000 UV-Vis sprectrophotometer (Thermo Scientific) by measuring absorbance at 260/280 nm. In order to convert mRNA to cDNA, reverse transcriptase PCR was performed using the high capacity cDNA reverse transcription kit (Applied Biosystems) according to the manufacturer’s protocol. Quantitative PCR (qPCR) was carried out using the ViiA^TM^ 7 Real-Time PCR system and Taqman universal PCR master mix (Applied Biosystems) in order to measure specific gene expression. The total reaction volume was 20 µl containing (100 ng cDNA +10 µl Taqman mastermix +1 µl Tagman endogenous control +1 µl Taqman gene expression). The primers/probes for GAPDH (Hs02786624_g1) and beta actin (ACTB) (Hs01060665_g1) were ordered from Thermo Fisher using the best coverage primer/probe set. Comparative Ct mode was used to analyze the qPCR samples and the runs consisted of temperatures at 50 °C for 2 minutes, 95 °C for 10 minutes, 40 cycles of 95 °C for 15 seconds and 60 °C for 1 minute. All qPCR samples were tested in biological triplicates. The expression of GAPDH mRNA was normalized with beta-actin mRNA expression and presented relative to the control sample GAPDH mRNA.

### LNPs delivered to Caco-2 monolayers

For Caco-2 monolayer experiments, Caco-2 cells (200,000 cells/well) were grown on BioCoat^TM^ HTS 1.0 µm filter supports as described previously^59^. Briefly, the cells were incubated in BSM for 2 days and EDM for 1 day. Both the BSM and EDM were supplemented with MITO+^TM^ Serum Extender according to the manufacturer’s protocol. The transepithelial electrical resistance (TEER) was measured with a Millicell Voltohmmeter to confirm the existence of a viable monolayer. Only Caco-2 monolayers with TEER values above 300 Ωcm^2^ were used for experiments. LNPs were formulated with Cy5.5-labeled siRNA at 0.3 mg/mL siRNA concentration. Caco-2 monolayers received LNPs with Cy5.5-labeled siRNA at an siRNA dose of 2000, 1000, 400, or 100 nM for up to 3 hours. After which, the monolayers were carefully removed and stained as described below.

### Animal Studies

Animal protocols were approved by the institutional animal care and use committee at Carnegie Mellon University (Pittsburgh, PA). All animal experiments were conducted in accordance with approved protocols. Female C57BL/6 mice of at least 6 weeks of age were purchased from Charles River Laboratories. Mice were housed under controlled temperature (25 °C) in 12 hour light-dark cycles. Animals were given access to standard diet and water.

### *In vivo* oral and rectal LNP gene silencing

Mice were orally or rectally gavaged with LNPs loaded with siGAPDH at a dose of 5 mg/kg. After 24 hours, mice were sacrificed and the colons were removed. Feces was flushed from the mouse colon using PBS in a syringe. The colons were carefully cut open to reveal the intestinal epithelium and intestinal cells were collected by scraping the insides of the colon with a spatula. For this experiment intestinal cells were collected from a 3 cm section above the mouse rectum. The intestinal epithelium was then placed in TRIzol® Reagent (Invitrogen) for mRNA extraction according to the manufacture’s protocol. Quantitative PCR was carried out as described above.

### *In vivo* oral delivery imaging studies

LNPs with Cy5.5-labeled siRNA were formulated as previously described at 0.05 mg/mL siRNA. Female C57BL/6 mice were orally gavaged with either PBS, Cy5.5-labeled siRNA, or LNPs containing Cy5.5-labeled siRNA at an siRNA dose of 0.5 mg/kg. The mice were fasted 4 hours prior to oral gavage. At specific time points after oral gavage (0.5, 1, 2, 4, 6, or 8 hours), the mice were sacrificed and organs removed for fluorescence imaging on an IVIS spectrum imaging system (Perkin Elmer, MA) at excitation and emission wavelengths of 675 and 720 nm. Images from the organs of each mouse were taken and the fluorescence was analyzed using Living Image^®^ software. The total radiant efficiency in ([p/s]/[µW/cm²]) was normalized across images.

### Confocal microscopy

For *in vivo* experiments, mice were orally gavaged with LNPs containing Cy5.5-labeled siRNA (ex:675/em:694). Six hours later, intestinal sections were excised from the jejunum of the small intestine and below the cecum in the colon. Tissues were fixed in 4% formaldehyde for 24 hours and washed three times with PBS. DNA and actin were stained with Hoescht 33342 (ex:350/em:461; 1:2,000 dilution) and Alexa Fluor 488 phalloidin (ex:495/em:518; 1:80 dilution) from Thermo Fisher for three hours. In some experiments, Caco-2 cells were stained with Alexa Fluor 594 ZO-1 monoclonal Antibody (ex:590/em:617; 1:100 dilution) for one hour. The samples were washed three times with PBS and imaged on a Zeiss LSM 700 confocal microscope.

### Data availability

Raw data will be made available upon reasonable request to the corresponding author.
